# Type of the recurrent exotropia after bilateral rectus recession for intermittent exotropia

**DOI:** 10.1186/s12886-016-0270-9

**Published:** 2016-07-08

**Authors:** Kwan Hyuk Cho, Hee Weon Kim, Dong Gyu Choi, Joo Yeon Lee

**Affiliations:** Department of Ophthalmology, Hallym University College of Medicine, Hallym University Sacred Heart Hospital, 896 Pyeongchon-dong, Dongan-gu, Anyang, Gyeonggi-do 431-070 South Korea; Department of Ophthalmology, Seoul National University College of Medicine, Seoul National University Bundang Hospital, Seongnam, South Korea; Department of Ophthalmology, Hallym University College of Medicine, Kangnam Sacred Heart Hospital, Seoul, South Korea

**Keywords:** Exotropia type, Distance-near difference, Recurrent exotropia, Bilateral rectus recession

## Abstract

**Background:**

The aim of this study was to investigate the type of exotropia (XT) based on the distance-near (D/N) difference in recurrent XT after bilateral lateral rectus (BLR) recession to treat intermittent XT (IXT) to look into the possibility of secondary convergence insufficiency (CI)-type strabismus.

**Methods:**

A total of 121 patients with recurrent XT after BLR recession for basic-type and divergence excess (DE)-type IXT were retrospectively enrolled at a single institution. The distributions in the XT types were compared according to the D/N difference between primary and recurrent XT.

**Results:**

Preoperatively, the population comprised 14 divergence excess (DE) types and 107 basic types. After the BLR recession, the XT-type composition changed to 59 basic types, 33 CI types, and 29 DE types. In one of the 14 preoperatively identified DE-type XT cases, the XT type changed to CI type, it changed to basic type in four cases, and the remaining nine cases showed no change in the DE type. The 107 preoperatively identified basic-type XT cases postoperatively became 55 basic-type, 32 CI-type, and 20 DE-type recurrent XT cases, and their postoperative distance XT control grades and near stereoacuity values did not significantly differ.

**Conclusion:**

The XT type composition changed after the BLR recession. The XT types in recurrent XT after BLR recession showed an increasing proportion of CI-type. We suspect that an individual fusion mechanism might also influence the XT-type in recurrent XT in view of the somewhat increased DE-type in recurrent XT.

## Background

Bilateral lateral rectus (BLR) recession is one of the best treatment options for intermittent exotropia (IXT), along with unilateral recess-resect surgery (R&R) [1–3]. Based on the concept that the therapies produce different effects on the distance/near (D/N) deviation, R&R has been classically recommended for basic-type exotropia (XT), and BLR recession, for divergence excess (DE)-type XT [4–7]. According to the concept, BLR recession has a greater corrective effect on distance than near deviation, bilateral medial rectus (BMR) resection has a greater corrective effect on near than distance deviation, and R&R has equal corrective effects on distance and near deviations depending on the mechanics of the eye movement in which resection of the medial rectus (MR) would strengthen convergence, and recession of the lateral rectus (LR) would weaken divergence [4–7]. Though these differences remain unproven, these mechanisms have been considered in the selection of a surgical method of treating XT as mentioned above [4–10].

Studies disagree on the superior surgical treatment method for IXT. In the classical studies conducted by Burian [7] and Kushner [8], R&R is the preferred method for basic-type XT, and BLR recession is preferred for divergence excess (DE)-type XT. In contrast, other studies reported good results for both basic- and DE-type XT treated using BLR recession [9–14]. Choi et al. [3] compared the long-term surgical outcomes of BLR recession and R&R in the basic-type intermittent exotropia, and found that they were better in the eyes treated using BLR recession than in those treated with R&R.

In addition to the good surgical outcomes, BLR recession also has several advantages. The procedure is short and simple; and unlike R&R, a larger amount of surgery procedures can be performed with BLR recession without causing symptomatic asymmetrical lateral incomitance [15–18]. Asymmetrical lateral incomitance after a maximal or supramaximal monocular R&R is known for the risk of symptomatic diplopia in ipsilateral side-gaze. The large LR recession can also cause lateral incomitance, but bilateral lateral incomitance is generally asymptomatic, particularly when symmetrical. Wright & Strube said that BLR recessions work well for all the basic, pseudo DE, and true DE types of XT, and is usually preferred over a monocular R&R as R&R produce significant lateral incomitance in the side of the operated eye [16].

However, it is deemed possible for the secondary convergence insufficiency (CI) type of IXT to be induced after BLR recession if the BLR recession affects the distance deviation rather than the near deviation. The purpose of this study was to evaluate the type of exotropia (XT) based on the distance-near (D/N) difference in recurrent XT after bilateral lateral rectus (BLR) recession for intermittent XT (IXT) to assess the possibility of secondary CI-type strabismus.

## Methods

The patients who underwent BLR recession for basic-type and DE-type IXT between January 1, 2003 and February 28, 2012 were retrospectively identified. Among them, the patients postoperatively diagnosed with recurrent XT with a distance angle greater than 10 prism diopters (PDs) were enrolled in this study. The cases of recurrent exotropia which occurred in patients who had experienced early surgical success were eligible, and we excluded the patients with early surgical failure. During the early postoperative period, we routinely followed-up the patients on the postoperative first day, first week, second week and first month after the surgery. Early surgical failure was defined as an eye alignment of ≥ 10 PD of distant angle of exotropia on the immediate postoperative period between the postoperative first day and second week. The patients who showed consecutive esotropia (≥5PD of distant angle of esotropia on the postoperative first month) were also excluded. The patients who had amblyopia, a high AC/A ratio, vertical transposition of the horizontal muscle during IXT surgery, or a systemic anomaly such as a neurologic disorder were excluded from this study.

All the procedures in this study conformed to the Declaration of Helsinki, and this study was approved by the Institutional Review Board (IRB) of the Hallym University Sacred Heart Hospital (IRB No. 2013-I025) with an understanding on exemption from the informed consent for the study of retrospective collection of the clinical data.

A single surgeon performed all the surgeries under general anesthesia. The written informed consent of all the patients and their legal guardians to the surgical procedure was obtained. The angle of deviation was determined through the alternate prism cover test, with accommodative targets for fixation at the distances of 6 m and 0.33 m.

The target angle at the time of the surgery was measured at a distance of 6 m and determined by performing a 1h occlusion on the non-dominant eye, and an alternate prism cover test before the patients were allowed to see using both eyes. The surgeon assessed and recorded the fusion control grade of the exodeviation (FCGX) at a fixation target of 6 m. The FCGX data collected from the medical records were graded as good or poor at a fixation target of 6 m. The FCGX was graded good when the patient manifested the deviation after the cover test and resumed the fusion rapidly with blinking or with a refixation. The patients who exhibited the deviation spontaneously, without any form of fusion disruption, were defined as having had a poor FCGX [19–21].

The type change between the primary XT and recurrent XT was evaluated according to the D/N deviation angles. The IXT type was assigned based on the same reference D/N difference in both of primary and recurrent exotropia. In the patients with a distance deviation greater than 30 PD, the reference value of the D/N difference was 10 PD; and in the patients with a distance deviation less than 30 PD, the reference value was one-third the distance deviating angle. Then the recurrent XT was classified into three IXT types: (1) the CI type, in which the near deviation exceeded the distance deviation by the reference value or greater; (2) the basic type, in which the D/N deviations were equal, or the D/N difference was within the reference value; and (3) the DE type, in which the distance deviation exceeds the near deviation by the reference value or greater.

The pseudo-DE-type IXT is generally differentiated from true DE type by the occlusion test. After the occlusion test, the near deviation increases and it becomes basic type. Meanwhile, IXT with a high AC/A ratio can be diagnosed when the near deviation increases in the +3.00 D lens test performed in the patient who has first undergone the monocular occlusion test to eliminated TPF. [4, 8, 19] In this study, we excluded the cases having a high AC/A ratio that were identified with the +3.00 D lens test made after the 1h occlusion test and before the patients were allowed to see with both eyes. The pseudo-DE type was preoperatively differentiated from the DE type in all the patients using the patch test, but the test was not performed in the patients with recurrent XT [8, 19]. To compare the cases of primary and recurrent XT, nine patients with preoperative pseudo-DE-type XT were assigned to the DE-type group, even though the pseudo-DE type is actually a subset of the basic type [4, 7].

The distributions of the XT types were compared based on the D/N difference between primary XT and recurrent XT. Also, we categorized the cases of preoperative basic-type XT into the following three groups according to the change in the XT type: the preoperative basic type to the basic type-recurrent XT (Basic-to-Basic group); the preoperative basic type to the CI-type recurrent XT (Basic-to-CI group); and the preoperative basic type to the DE-type recurrent XT (Basic-to-DE group). To determine the relevant preoperative clinical factors, the Basic-to-CI group and the Basic-to-DE group were compared with the Basic-to-Basic group. The postoperative sensory-motor outcomes were also compared according to the recurrent XT type in the three groups. The postoperative clinical data used in the analysis were based on the most recent medical record of each patient after the BLR recession. This categorization could not be performed in the preoperative DE-type XT because there were too few patients and there was no discrimination between the pseudo-DE and true DE types.

Multiple logistic regression analysis was used to examine the influence of the preoperative factors on the XT type change in the three groups. The chi-square test was used to compare the postoperative outcomes of the three groups. A probability value of < 0.05 was considered statistically significant. All the analyses were performed using SPSS version 12.0.0 for Windows (SPSS Inc., Chicago, IL, USA).

## Results

One hundred twenty-one patients with recurrent XT were included in this study. Sixty-one of them were male, and their mean age at their surgery was 8.92 ± 7.11 years. The mean follow-up period after the surgery was 3.74 ± 2.48 years, and the mean length of time from the surgery to the recurrence was 1.44 ± 1.80 years. The mean preoperative distance deviation angle was 27.54 ± 9.75 PD, and the mean near-deviation angle was 26.22 ± 11.71 PD (Table 1). The mean postoperative distance deviation angle in the cases of recurrent XT was 15.01 ± 4.81 PD, and the mean near-deviation angle was 15.69 ± 8.91 PD.

**Table 1:** Proportions of the preoperative exotropia type and the deviation angle

XT type	N	Deviation angle (Distance/Near)^a^	Follow-up (Year ± SD)
Basic	107	27.54 ± 10.23/28.30 ± 10.66	3.67 ± 2.29
DE	14	27.64 ± 5.54/11.00 ± 7.70	4.22 ± 2.72
Total	121	27.54 ± 9.75/26.22 ± 11.71	3.74 ± 2.48

^a^“Distance” indicates exotropia at 6 m; “near” indicates exotropia at 0.33 m; the values are in prism diopters, mean ± SD; *XT* exotropia; *CI* convergence insufficiency, and *DE* divergence excess

Preoperatively, the population comprised 107 basic types and 14 DE types (Table 1). After the BLR recession, the XT type composition changed to 59 basic types, 33 CI types, and 29 DE types. The XT type changed from primary XT to recurrent XT in 57 patients. In the 107 preoperative basic types, 32 patients converted to the CI type, and 20 patients, to the DE type. In the 14 preoperative DE types, four patients converted to the basic type, and one patient, to the CI type. These 5 patients were all pseudo-DE type XT and all three of the true DE type XT maintained DE type in recurrent exotropia (Table 2).

**Table 2:** Exotropia type change in recurrent exotropia after bilateral lateral rectus (BLR) recession

Exotropia type in primary exotropia (N)	Exotropia type In recurrent exotropia (N)	Deviation angle in primary exotropia (Distance/Near)^a^	Deviation angle in recurrent exotropia (Distance/Near)^a^
Basic (107)	DE^b^ (20)	30.2/29.9	14.3/5.4
	Basic (55)	26.2/26.3	15.3/24.9
	CI (32)	27.5/29.7	17.1/17.5
Pseudo-DE (11)	DE^b^ (6)	28.3/12.0	13.7/2.0
	Basic (4)	29.3/11.8	15.5/15.8
	CI (1)	25.0/15.0	12.0/20.0
True-DE (3)	DE^c^ (3)	25.0/4.0	16.7/6.0
	Basic (0)	-	-
	CI (0)	-	-

^a^“Distance” indicates exotropia at 6 m; “near” indicates exotropia at 0.33 m; the values are in prism diopters, mean; ^b^“DE” indicates presumed pseudo-divergence excess type of recurrent exotropia which was not confirmed for the lack of occlusion test in recurrent exotropia; ^c^“DE” indicates true-divergence excess type which persisted from primary exotropia to recurrent exotropia, *CI* convergence insufficiency, and *DE* divergence excess

**Fig. 1:**
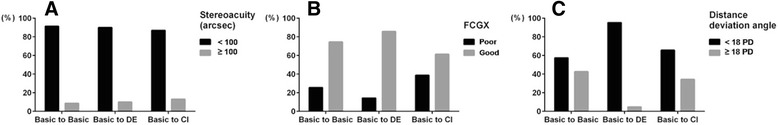
Bar graph showing the postoperative sensory-motor outcomes according to the postoperative type change in recurrent exotropia from the preoperative basic-type exotopia. **a** Stereoacuity: Nearly all the patients in the three groups had a good stereoacuity (*P* = 0.116). **b** Fusion control grade of the exodeviation (FCGX): The FCGX did not differ significantly across the three groups (*P* = 0.254). **c** Distance deviation angle: The DE-type recurrent exotropia group (Basic-to-DE group) had a significantly smaller angle deviation than the other groups (*P* = 0.007)

The analysis of the 107 patients with preoperative basic-type IXT showed that the preoperative clinical factors, including the FCGX, distance deviation angle, stereoacuity, use of corrective glasses, and age at surgery, did not affect the change in the XT type in the Basic-to-CI group and the Basic-to-DE group (Tables 3 and 4). Likewise, the mean surgical numbers of the recession amount and the mean length of time from the surgery to the recurrence did not differ in the three groups (Table 5). The postoperative sensory-motor outcomes, including the stereoacuity, FCGX, and distance deviation angle, of the three groups were compared. The stereoacuity and FCGX did not differ across the groups (*P* = 0.116, Fig. 1a, *P* = 0.254, Fig. 1b). The distance deviation angle was greatest in the Basic-to-Basic group and least in the Basic-to-DE group (P = 0.007, Fig. 1c).

**Table 3:** Univariate analysis identifying the preoperative clinical factors that affected the exotropia type changes

Preoperative factors	Basic to basic group (*N* = 55)	Basic to CI group (*N* = 32)	*P*-value^a^	Basic to DE group (*N* = 20)	*P*-value^b^
FCGX (good : poor) (N)	16:38	13:19	0.349^c^	3:18	0.241^c^
Distance deviation angle (PD)	26.2	27.5	0.474^d^	30.2	0.095^d^
Age at surgery (yrs)	8.5	7.4	0.514^d^	13.2	0.513^d^
Stereoacuity (sec)	151.6	91.1	0.213^d^	141.8	0.876^d^
Corrective glasses (N)	36	24	0.474^c^	15	0.787^c^

*P*-value^a^ = Basic to Basic group versus Basic to CI group ; *P*-value^b^ = Basic to Basic group versus Basic to DE group; *FCGX* fusion control grade of the exodeviation, *CI* convergence insufficiency, *DE* divergence excess; and *PD* prism diopters

^c^Fisher’s exact test, ^d^Wilcoxon signed rank test, *P* < 0.05 significant

**Table 4:** Multiple logistic regression analysis identifying the preoperative clinical factors that affected the type changes

Preoperative factor	Basic to CI group			Basic to DE group		
	*P*-value	OR	95 % C.I.	*P*-value	OR	95 % C.I.
FCGX	0.471	0.759	0.358–1.607	0.177	2.342	0.681–1.058
Distance deviation angle	0.427	1.024	0.486–1.028	0.109	1.040	0.645–1.547
Age at surgery	0.126	0.942	0.872–1.017	0.754	1.000	0.997–1.002
Stereoacuity	0.130	0.997	0.992–1.001	0.823	0.881	0.290–2.673
Corrective glasses	0.449	0.685	0.258–1.823	0.406	1.021	0.973–1.017

*P* < 0.05 significant, compared to the Basic-to-Basic group; *OR* odds ratio, *C.I.* confidence interval, *FCGX* fusion control grade of the exodeviation

**Table 5:** Characteristics of the three groups according to the change in the exotropia type after surgery

	Basic to basic group (*n* = 55)	Basic to CI group (*n* = 32)	Basic to DE group (*n* = 20)	*P* Value
Follow-up to recurrence (years)	1.36 ± 1.90 (0.1–9)	1.64 ± 1.98 (0.1–7)	1.34 ± 1.60 (0.1–6)	0.770
Last follow-up (years)	3.40 ± 2.31 (0.5–9)	4.20 ± 2.70 (0.5–10)	3.52 ± 2.45 (0.5–8)	0.337
BLR recession amount (mm)	6.25 ± 1.30 (4–11)	6.56 ± 1.66 (5–12)	6.90 ± 2.31 (5–12)	0.302

*Basic* basic type, *CI* convergence insufficiency type, *DE* divergence excess type

## Discussion

IXT types are commonly classified into three groups of basic, DE and CI type, based on the difference between the D/N deviations [6, 7, 22]. The reference value of the D/N differences used in the classification differs across studies. Hardesty et al. [13] relied on a 5-PD D/N difference; Burian and Spivey [6, 7] recommended a 10PD difference; and other researchers have relied on a 15PD value [22]. We used the reference value of the D/N differences described by Suh et al. [23] to avoid underestimating the change in the XT type in cases of recurrent XT with a low-grade distance angle. In cases with a distance angle greater than 30 PD, the reference value of the D/N difference was 10 PD; and in cases with a distance angle less than 30 PD, the reference value was one-third of the distance angle.

The classic concept hypothesized that BLR recession selectively affects the distance deviation more than the near deviation [6, 7]. As mentioned previously, many recent studies generally demonstrated good results after BLR recession [3, 9–13]. However, most previous intermittent XT studies were confined to the surgical success and recurrence rate, and few studies assessed the postoperative D/N differences after these XT surgeries [3, 7, 9–13]. We’re concerned about the possibility of secondary convergence insufficiency (CI)-type strabismus. It could be a downside of the BLR recession because the CI type requires delicate management among the three IXT types [24–27], and headache, diplopia, blurred vision, and asthenopia had reportedly accompanied it [4, 7, 25]. According to our results, the XT type composition changed after the BLR recession for basic- and DE-type IXT and it showed an increasing proportion of CI-type XT.

29.9 % (32/107) of patients got the CI-type recurrent IXT from the preoperative basic-type IXT. There were no preoperative factors that affect the occurrence of CI type in the basic-type preoperative XT (Tables 3 and 4). In the analysis of the postoperative motor-sensory outcomes, stereoacuity and FCGX did not significantly differ between the three groups in the basic-type preoperative XT. This indicates that most of the recurrent XT cases showed a good fusional state regardless of the XT type. That is, BLR recession did not weaken the near fusion mechanism though the distance XT was more affected than the near exodeviation in the Basic-to-CI group.

Twenty cases out of the 107 preoperative Basic-type XT were also converted into the DE type. However, we think that the Basic-to-DE groups didn’t experience significant type change. We did not perform a patch test to differentiate the pseudo-DE type from the true DE type in the recurrent XT cases. Although we did not confirm the cases using the patch test, recurrent DE-type XTs in Basic-to-DE group are actually pseudo-DE-type XTs with the tenacious proximal fusion (TPF) mechanism because a true DE type has inherent nature with unique features of large proximal convergence [4, 7] and there was a bare possibility that it was newly occurred postoperatively. Besides, many patients had a relatively small distance deviation angle in the Basic-to-DE group, even though there were no differences in time to the recurrence or total follow-up period between the three groups (Table 5). We suggested the Basic-to-DE group didn’t demonstrate XT type change and it was a group representing good surgical outcome. But, the Basic-to-CI group didn’t showed inferior surgical outcomes in the distance deviation angle, FCGX, and near stereopsis when compared to the Basic-to-Basic group and had the similar length of time to the recurrence and similar follow-up time to the other groups. We concluded the type change in the Basic-to-CI group could be surgically induced and the Basic-to-CI group was not merely a group of poor postoperative state.

Also, only one CI-type XT (7.1 %) was occurred in the 14 preoperative DE-type XTs. It is thought that a strong near fusion mechanism may maintain the near deviation in DE-type XT. This finding could support the hypothesis of Kushner that patients with the TPF mechanism fare better after LR recession than patients without the TPF mechanism [8].

In the results, there were another four patients they showed a difference in IXT types between their primary XT and recurrent XT. They got basic-type recurrent IXT from the preoperative DE type. But all four patients were actually pseudo-DE type (a subset of basic type) preoperatively and we also think it was not a real change.

Finally, the incidence of secondary CI type in our results were 7.1 % (1/14) in preoperative DE type and 29.9 % (32/107) in preoperative basic type after BLR recession. Our results showed a certain risk in occurrence of secondary CI-type IXT, especially in the basic type-XT with lack of TPF mechanism. To confirm this, direct comparison of BLR recession and R&R in a large number of patients will be needed.

As far as we know, this is the first study that compared the distribution of the XT types according to the D/N difference between primary and recurrent XT after BLR recession to find out the possibility of secondary CI-type IXT. Limitation of this study is that it was a single-center study, which collected data retrospectively. Future studies should be contemplated as multicenter and prospective studies in a large number of patients. Another limitation of this study is that the DE-type category included both the pseudo-DE and true DE types and we did not perform a patch test to differentiate the pseudo-DE type from the true DE type in the recurrent XT cases, as mentioned above.

## Conclusions

In conclusion, The XT type composition changed after BLR recession for basic-type and DE-type IXT. The XT types in recurrent exotropia after BLR recession showed an increasing proportion of CI-type XT. Fortunately, it did not significantly disrupt the stereopsis or motor fusion control.

## Abbreviations

BLR, bilateral lateral rectus; BMR, bilateral medial rectus; CI, convergence insufficiency; D/N, distance-near; DE, divergence excess; FCGX, fusion control grade of the exodeviation; IXT, intermittent exotropia; LR, lateral rectus; MR, medial rectus; PDs, prism diopters; XT, exotropia

## References

[1] Nishimura J, Okino L (1999). Outcome study of bilateral lateral rectus recession for intermittent exotropia in children. Ophthalmic Surg Lasers.

[2] Stoller SH, Simon JW, Lininger LL (1993). Bilateral lateral rectus recession for exotropia: a survival analysis. J Pediatr Ophthalmol Strabismus.

[3] Choi J, Chang JW, Kim SJ, Yu YS (2012). The Long-term Survival Analysis of Bilateral Lateral Rectus Recession versus Unilateral Recession-Resection for Intermittent Exotropia. Am J Ophthalmol.

[4] Kushner BJ, Morton GV (1998). Distance/Near Differences in Intermittent Exotropia. Arch Ophthalmol.

[5] Burian HM, Spivey BE (1964). The Surgical Management of Exodeviations. Trans Am Ophthalmol Soc.

[6] Burian HM, Spivey BE (1965). The Surgical Management of Exodeviations. Am J Ophthalmol.

[7] Burian HM (1966). Exodeviations: Their Classification, Diagnosis, and Treatment. Am J Ophthalmol.

[8] Kushner BJ (1998). Selective Surgery for Intermittent Exotropia based on Distance/Near Differences. Arch Ophthalmol.

[9] Kushner BJ (1999). Diagnosis and Treatment of Exotropia with a High Convergence-Accommodation Ratio. Arch Ophthalmol.

[10] Keenan JM, Willshaw HE (1994). The Outcome of Strabismus Surgery in Childhood Exotropia. Eye (Lond).

[11] Parks MM (1955). Divergent Strabismus. Clin Proc Child Hosp Dist Columbia.

[12] Lee SY, Lee YC (2001). Relationship between Motor Alignment at Postoperative Day 1 and at Year 1 after Symmetric and Asymmetric Surgery in Intermittent Exotropia. Jpn J Ophthalmol.

[13] Hardesty HH, Boynton JR, Keenan JP (1978). Treatment of Intermittent Exotropia. Arch Ophthalmol.

[14] Yam JC, Wu PK, Chong GS, Wong US, Chan CW, Ko ST (2012). Long-term ocular alignment after bilateral lateral rectus recession in children with infantile and intermittent exotropia. J AAPOS.

[15] Celebi S, Kükner AS (2001). Large Bilateral Lateral Rectus Recession in Large-Angle Divergence Excess Exotropia. Eur J Ophthalmol.

[16] Wright KW, Strube YNJ (2012). Pediatric Ophthalmology and Strabismus.

[17] Schwartz RL, Calhoun JH (1980). Surgery of large angle exotropia. J Pediatr Ophthalmol Strabismus.

[18] Currie ZI, Shipman T, Burke JP (2003). Surgical correction of large-angle exotropia in adults. Eye (Lond).

[19] Rosenbaum AL, Santiago AP (1999). Intermittent Exotropia: Clinical Strabismus Management.

[20] Haggerty H, Richardson S, Hrisos S, Strong NP, Clarke MP (2004). The Newcastle Control Score: a new method of grading the severity of intermittent distance exotropia. Br J Ophthalmology.

[21] Yam JC, Chong GS, Wu PK, Wong US, Chan CW, Ko ST (2013). A prospective study of fusional convergence parameters in Chinese patients with intermittent exotropia. J AAPOS.

[22] Von Noorden GK (1969). Divergence Excess and Simulated Divergence Excess: Diagnosis and Surgical Management. Doc Ophthalmol.

[23] Suh YW, Kim SH, Lee JY, Cho YA (2006). Conversion of Intermittent Exotropia Types Subsequent to Part-time Occlusion Therapy and Its Sustainability. Graefe’s Arch Clin Exp Ophthalmol.

[24] Snir M, Axer-Siegel R, Shalev B, Sherf I, Yassur Y (1999). Slanted Lateral Rectus Recession for Exotropia with Convergence Weakness. Ophthalmology.

[25] Hermann JS (1980). Surgical Therapy of Convergence Insufficiency. J Pediatr Ophthalmol Strabismus.

[26] Biedner B (1997). Treatment of Convergence Insufficiency by Single Medial Rectus Muscle Slanting Resection. Ophthalmic Surg Lasers.

[27] Kushner BJ (2005). The Treatment of Convergence Insufficiency. Arch Ophthalmol.

